# Quercetin Prevents Pyrrolizidine Alkaloid Clivorine-Induced Liver Injury in Mice by Elevating Body Defense Capacity

**DOI:** 10.1371/journal.pone.0098970

**Published:** 2014-06-06

**Authors:** Lili Ji, Yibo Ma, Zaiyong Wang, Zhunxiu Cai, Chun Pang, Zhengtao Wang

**Affiliations:** 1 The MOE Key Laboratory for Standardization of Chinese Medicines, The SATCM Key Laboratory for New Resources and Quality Evaluation of Chinese Medicines and Shanghai Key Laboratory of Complex Prescription, Institute of Chinese Materia Medica, Shanghai University of Traditional Chinese Medicine, Shanghai, China; 2 Shanghai R&D Centre for Standardization of Chinese Medicines, Shanghai, China; University of Louisville School of Medicine, United States of America

## Abstract

Quercetin is a plant-derived flavonoid that is widely distributed in nature. The present study is designed to analyze the underlying mechanism in the protection of quercetin against pyrrolizidine alkaloid clivorine-induced acute liver injury *in vivo*. Serum transaminases, total bilirubin analysis, and liver histological evaluation demonstrated the protection of quercetin against clivorine-induced liver injury. Terminal dUTP nick end-labeling assay demonstrated that quercetin reduced the increased amount of liver apoptotic cells induced by clivorine. Western-blot analysis of caspase-3 showed that quercetin inhibited the cleaved activation of caspase-3 induced by clivorine. Results also showed that quercetin reduced the increase in liver glutathione and lipid peroxidative product malondialdehyde induced by clivorine. Quercetin reduced the enhanced liver immunohistochemical staining for 4-hydroxynonenal induced by clivorine. Results of the Mouse Stress and Toxicity PathwayFinder RT^2^ Profiler PCR Array demonstrated that the expression of genes related with oxidative or metabolic stress and heat shock was obviously altered after quercetin treatment. Some of the alterations were confirmed by real-time PCR. Our results demonstrated that quercetin prevents clivorine-induced acute liver injury *in vivo* by inhibiting apoptotic cell death and ameliorating oxidative stress injury. This protection may be caused by the elevation of the body defense capacity induced by quercetin.

## Introduction

Pyrrolizidine alkaloid (PA) is a type of natural hepatotoxin and widely distributed in thousands of plants worldwide [1], [2]. In Europe, South/North America, Japan, China, etc., PA intoxication of humans and livestocks via the consumption of traditional herbs, tonic prescription, tea, or other PA-contaminated foods, including milk, honey, or meat, have been reported [3]. PA-induced hepatotoxicity has been extensively investigated. However, no antidote has been developed for the PA-induced hepatotoxicity. The US FDA and British Medicines Healthcare Products Regulatory Agency proposed a series of research programs, instructions, and standards to alert people on the hepatotoxicity of PA and its related herbs and remedies.

Clivorine, an otonecine-type PA abundant in *Ligularia hodgsonii* Hook and *Ligularia Dentata*, have been generally used to treat cough, hepatitis, and inflammation in traditional Chinese medicine [4], [5]. *L. hodgsonii* Hook and *L. Dentata*, which belong to genus *Ligularia* and the family Asteraceae, are native to China and Japan [6]. We already demonstrated in our previous studies the involvement of oxidative stress injury and apoptosis in clivorine-induced hepatotoxicity [7], [8]. N-Acetyl-Cysteine and S-adenosyl methionine, which are the precursors for cellular GSH biosynthesis, can reverse clivorine-induced hepatotoxicity by increasing cellular GSH level [9], [10]. Previous studies have indicated that antioxidant compounds may have potential protective action against PA-induced liver injury.

Flavonoids, a large group of natural polyphenolic compounds, are powerful antioxidants found in various fruits, vegetables, tea, red wine, and medicinal herbs. Flavonoids can scavenge free radicals and other oxidizing intermediates because of their phenolic hydroxyl groups and thus contribute to the counteraction of body against a great variety of diseases [11]. Quercetin (3,3′,4′,5,7-pentahydroxyflavone) is one of the most abundant flavonoids and widely distributed in nature. Given its well-known antioxidant ability, quercetin exhibits therapeutic potential against various liver injuries caused by toxins, which include carbon tetrachloride, ethanol, thioacetamide, paracetamol, etc. [12]–[15]. The present study is designed to analyze the mechanism underlying the protection of quercetin against PA clivorine-induced acute liver injury.

## Materials and Methods

### Ethics Statements

All animal experiments were performed according to the protocol approved by the Experimental Animal Ethical Committee of Shanghai University of Traditional Chinese Medicine (Approved Number: 11002). All mice received humane care, and all efforts were made to minimize suffering.

### Chemical compounds and reagents

Clivorine was isolated from *L. hodgsonii* Hook and identified by IR, NMR, and MS with 99.5% purity [4]. Quercetin was purchased from Sigma Chemical Co. (St. Louis, MO). Terminal dUTP nick end-labeling (TUNEL) staining system (TdT-FragEL DNA Fragmentation Detection Kit) was purchased from Merck Calbiochem (Darmstadt, Germany). The kits for determining serum alanine/aspartate transaminase (ALT/AST) activity, and total bilirubin (TB) level were obtained from the Shanghai Rongsheng Biotech Corporation (Shanghai, China). Anti-caspase-3 antibody was purchased from Cell Signaling Technology (Danver, MA). Peroxidase-conjugated goat anti-rabbit immunoglobulin G (IgG) (H+L) was purchased from Jackson ImmunoResearch (West Grove, PA). BCA Protein Assay Kit was purchased from Thermo Scientific (Rockford, IL). Anti-4 Hydroxynonenal (4-NHE) antibody was purchased from Abcam (Cambridge, UK). The DAKO EnVision detection system was purchased from DAKO Corporation (Carpinteria, CA). Trizol reagent was purchased from Life Technology (Carlsbad, CA). PrimeScript® RT Master Mix and SYBR Premix Ex Taq were purchased from Takara (Shiga, Japan). RT^2^ Profiler PCR array was purchased from Qiagen (Hilden, German). All other reagents were purchased from Sigma (St. Louis, MO), unless otherwise indicated.

### Experimental animals

Specific pathogen-free male ICR mice (body weight: 16 g to 20 g) were purchased from Shanghai Laboratory Animal Center of Chinese Academy of Science (Shanghai, China). The mice were fed with standard laboratory diet and were given free access to tap water. The animal room was maintained at a temperature of 22±1°C with a 12 h light–dark cycle (6:00 to 18:00) and 65%±5% humidity.

### Treatment of animals

ICR mice were divided into six groups: (1) vehicle control, (2) clivorine (210 mg/kg), (3) clivorine (210 mg/kg) + quercetin (40 mg/kg), (4) clivorine (210 mg/kg) + quercetin (60 mg/kg), (5) clivorine (210 mg/kg) + quercetin (90 mg/kg), and (6) quercetin (90 mg/kg). The mice were pre-administered orally with quercetin for 7 consecutive days. After quercetin administration for 1 h on the last day, the mice were given a single dose of clivorine (210 mg/kg, i.g.). The mice were anesthetized with urethane 24 h after clivorine intoxication, the blood samples were taken from the abdominal aorta, and liver was collected.

### ALT, AST, and TB Assay

Fresh blood was obtained from mice and put at room temperature for 60 min to clot. After centrifugation at 3 000×*g* for 15 min, the serum was collected in new tubes. Serum ALT and AST activities, as well as TB level, were determined by kits according to the manufacturer's instructions.

### Histological observation

The liver samples were fixed in 10% PBS-formalin and embedded in paraffin. Samples were subsequently sectioned (5 µM), stained with hematoxylin and eosin, and examined under a light microscope (Olympus, Japan) to evaluate the liver damage.

### TUNEL assay

Apoptotic hepatocytes were labeled *in situ* using a TUNEL peroxidase apoptosis detection kit according to the manufacturer's instructions. An inverted microscope (Nikon, Japan) was used to observe apoptotic cells. A dark brown DAB signal indicated positive staining (apoptotic cells). Apoptotic hepatocytes were counted manually in at least three random fields using a light microscope at 100× magnification.

### Western-blot analysis

Liver tissues (approximately 40 mg) were homogenized in ice-cold lysis buffer containing 50 mM Tris (pH 7.5), 1 mM ethylenediaminetetraacetic acid (EDTA), 150 mM NaCl, 20 mM NaF, 0.5% NP-40, 10% glycerol, 1 mM phenylmethylsulfonyl fluoride, 10 µg/mL aprotinin, 10 µg/mL leupeptin, 10 µg/mL pepstatin A and centrifuged at 3000 g for 5 min before transferring the supernatant to new tubes. Protein concentrations were analysis by BCA method, and every sample was normalized to the equal protein concentration. Proteins were separated by SDS-PAGE and blots were probed with appropriate combination of primary and HRP-conjugated secondary antibodies.

### Measurement of tissue lipid peroxidation (LPO)

LPO in tissues was determined by a previously reported method [16]. Malondialdehyde (MDA) is an end product of LPO and generally serves as an index of the intensity of LPO. MDA reacts with 2-thiobarbituric acid to generate a pink-colored product, which has an absorbance at 532 nm. The tissue MDA level was calculated based on the tissue protein concentration.

### Measurement of liver GSH level

Liver GSH and GSSG levels were determined by the 5, 5-dithio-bis (2-nitrobenzoic acid) assay according to the reported method described in our previous published paper [17].

### Immunohistochemical Analysis of 4-HNE

Paraffin-embedded liver sections (5 µm thickness) were deparaffinized in xylene, and rehydrated in a gradient of ethanol to distilled water. After quenching endogenous peroxidase activity with 3% hydrogen peroxide, tissue sections were incubated with 5% bovine serum albumin to minimize nonspecific binding. Monoclonal 4-HNE antibody was applied to sections at 4°C overnight. The antigen-antibody reactions on sections were detected using DAKO EnVision detection kits. All sections were counterstained with hematoxylin. Images were taken using an inverted microscope (Nikon, Japan). The 4-HNE expression was determined by image information object definition (IOD) values analyzed by Image-Pro Plus 6.0 (Media Cybernetics, Silver Spring, MD).

### RNA isolation and cDNA synthesis

Liver total RNA was isolated using Trizol reagent according to the manufacturer's instruction. The RNA content was determined by measuring the optical density at 260 nm, and cDNA was synthesized according to the manufacturer's instruction described in PrimeScript® RT Master Mix kits.

### RT^2^ Profiler PCR array

Mouse Stress and Toxicity PathwayFinder RT^2^ Profiler PCR array was performed for quantitative PCR in the ABI 7900 system (Applied Biosystems, USA) with the following cycling conditions: 10 min at 95°C, 15 s at 95°C, and 1 min 60°C for 40 cycles with a final 4°C hold. Five endogenous control genes, namely, glucuronidase β, hypoxanthine guanine, heat-shock protein 90, glyceraldehyde phosphate dehidrogenase, and β-actin, found on the PCR array were used for data normalization. Each replicate cycle threshold (Ct) was normalized to the average Ct value of 5 endogenous controls per plate. Results were calculated using the 2 ^−ΔΔCt^ method. Heat map was generated using the web-based program of RT^2^ Profiler PCR Array Data Analysis. Variations in the liver gene expression between control and quercetin-treated animals are shown as a fold of increase or decrease.

### Real-time PCR analysis

Quantitative real-time PCR was used to further verify the mRNA expression levels of SOD1, SOD2, Hmox1, Hmox2, Fmo5, Ephx2, Polrk2, Cyp2b10, Cyp1b1, Cyp2a5, Cyp2b9, Cyp3a11, Cyp7a1, Hspa1l, Hspe1, Hspa1b, and Dnaja 1. Real-time PCR was performed with STEPONE Plus (Carlsbad, CA, USA) using a SYBR green premix according to the manufacturer's instructions. Relative expression of target genes was normalized to Actin, analyzed by 2 ^−ΔΔCt^ method and given as ratio compared with the control. The primer sequences used in this study are shown in Table S1.

### Statistical analysis

Data were expressed as means ± standard error of the mean. The significance of differences between groups was evaluated by one-way ANOVA with LSD post hoc test, and *P<0.05* was considered as statistically significant differences.

## Results

### Quercetin prevents clivorine-induced liver injury

**Figure 1:**
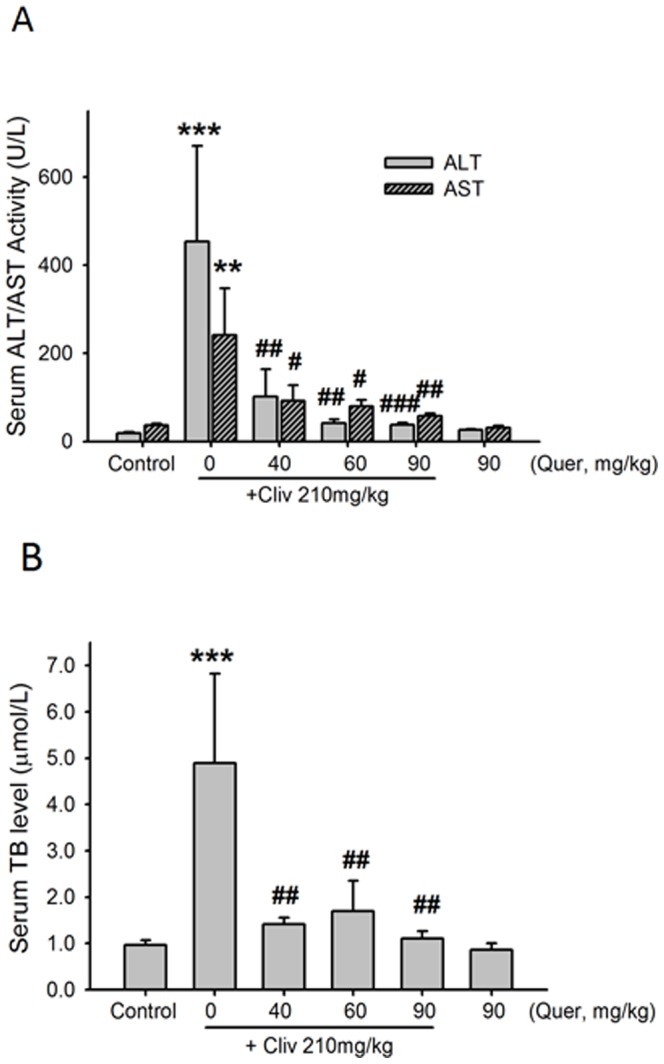
Quercetin reduced the clivorine-increased serum ALT/AST activity and TB level. (**A**) ALT/AST activity. (**B**) TB level. Data are expressed as means ± SEM (n = 9 to 10). **P<0.01, ***P<0.001 compared with the control; *^#^P<0.05, ^##^P<0.01, ^###^P<0.001* compared with clivorine. Cliv: Clivorine, Quer: Quercetin.

**Figure 2:**
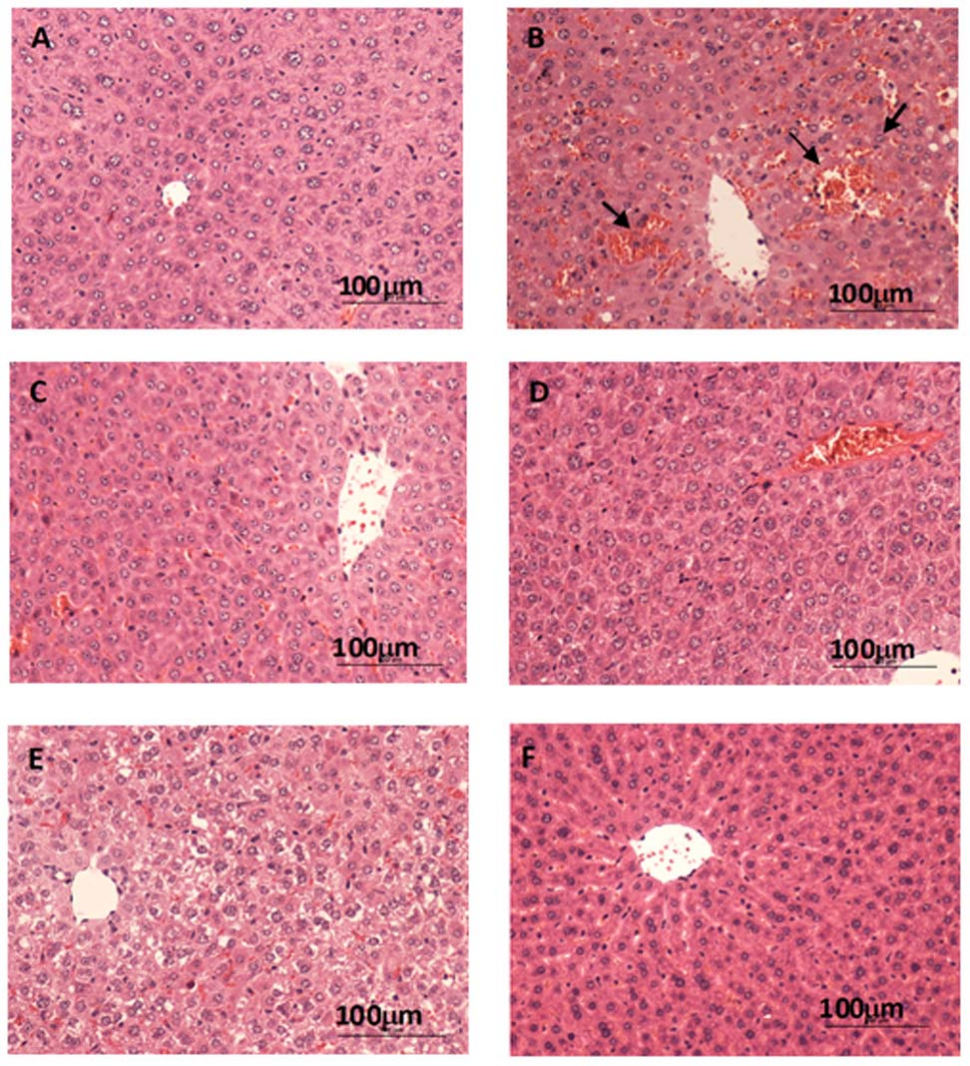
Histological observation of the protection of quercetin against clivorine-induced liver injury. After treatment, livers were removed, fixed, sectioned (5 µm), and processed for hematoxylin and eosin staining. Typical images were selected from each experimental group (original magnification: 100×). (**A**) Vehicle control, (**B**) clivorine (210 mg/kg), (**C**) clivorine (210 mg/kg) + quercetin (40 mg/kg), (**D**) clivorine (210 mg/kg) + quercetin (60 mg/kg), (**E**) clivorine (210 mg/kg) + quercetin (90 mg/kg), and (**F**) quercetin (90 mg/kg). Arrows indicate the intrahepatic hemorrhage.

Clivorine (210 mg/kg) obviously increased serum ALT and AST activity (P<0.01, P<0.001) (Fig. 1A), while quercetin (40, 60, and 90 mg/kg) reduced the clivorine-induced increase in these parameters (P<0.05, P<0.01, P<0.001). Clivorine increased serum TB level (P<0.001), but quercetin inhibited such increase (P<0.01) (Fig. 1B). Additionally, clivorine-treated mice showed severe liver damage, indicating intrahepatic hemorrhage and destruction of liver structure (Fig. 2B). Quercetin can reverse such liver injury in a dose-dependent manner (Figs. 2C to E). A liver histological picture of quercetin (90 mg/kg)-treated mice is shown in Fig. 2F, and no remarkable difference was found compared with control.

### Quercetin prevents clivorine-induced apoptosis in liver

**Figure 3:**
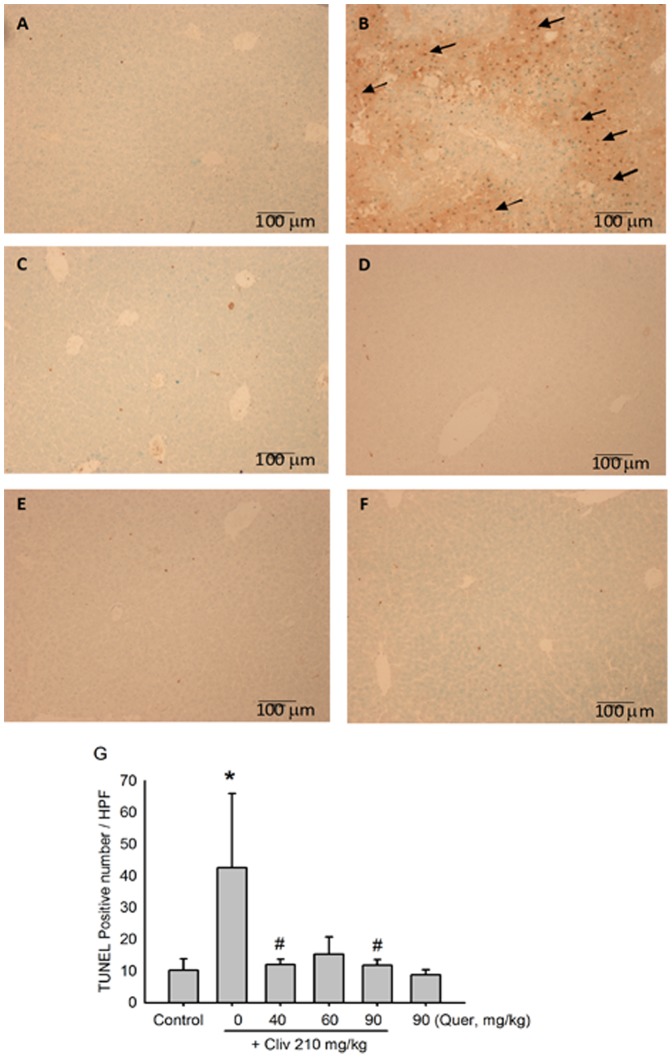
Hepatocyte apoptosis was evaluated via TUNEL staining assay. Typical images were selected from each experimental group (original magnification: 100×). (**A**) Vehicle control, (**B**) clivorine (210 mg/kg), (**C**) clivorine (210 mg/kg) + quercetin (40 mg/kg), (**D**) clivorine (210 mg/kg) + quercetin (60 mg/kg), (**E**) clivorine (210 mg/kg) + quercetin (90 mg/kg), and (**F**) Quercetin (90 mg/kg). Arrows indicate apoptotic hepatocytes. (**G**) The apoptotic hepatocytes were counted manually in at least three random fields every section. Data are expressed as means ± SEM (n = 6). *P<0.05 compared with the control; *^#^P<0.05* compared with clivorine.

**Figure 4:**
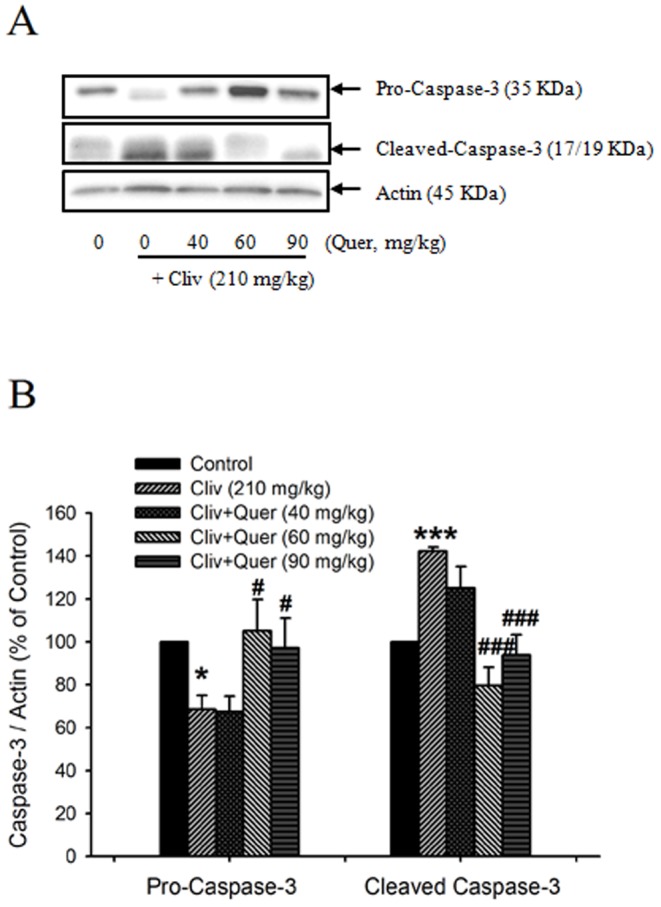
Quercetin inhibited clivorine-induced cleavage of caspase-3. (A) Protein expression of pro-caspase-3 (35 KDa) and cleaved caspase-3 (17/19 KDa) was determined by Western-blot analysis. The western blot figure represents one of at least three independent experiments with similar results. (B) The quantitative analysis of Western-blot results. Data were expressed as means ± SEM (n = 3 to 4). * P<0.05, *** P<0.001 compared with the control; *^#^ P<0.05, ^###^ P<0.001* compared with clivorine.

From the TUNEL assay, no apoptotic cells were found in the liver of the control mice (Fig. 3A). After clivorine administration (210 mg/kg), the liver demonstrated an increased amount of scattered TUNEL-positive apoptotic hepatocytes (Fig. 3B). Administration of various doses of quercetin reduced the TUNEL-positive apoptotic cells enhanced by clivorine (Figs. 3C to E). No apoptotic cells were found in the livers of quercetin (90 mg/kg)-treated mice (Fig. 3F). Apoptotic cell count showed that clivorine (210 mg/kg) obviously increased the number of apoptotic cells (P<0.05), while quercetin (40 and 90 mg/kg) decreased AP-increased apoptotic cell number (P<0.05) (Fig. 3G). Furthermore, Western-blot results (Fig. 4) showed that clivorine decreased the expression of pro-caspase-3 and increased the expression of cleaved caspase-3, which indicates that clivorine can induce the cleaved activation of caspase-3. Quercetin (60, 90 mg/kg) reversed the decreased expression of pro-caspase-3 and the increased expression of cleaved caspase-3 induced by clivorine. The inhibition of quercetin on the cleaved activation of caspase-3 induced by clivorine further evidences that quercetin can inhibit clivorine-induced liver apoptosis.

### Quercetin prevents clivorine-induced oxidative liver injury

**Figure 5:**
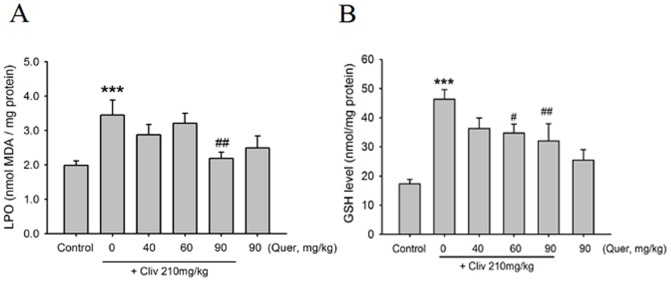
Liver GSH and MDA assay. (A) Effect of quercetin on clivorine-increased liver LPO by analyzing MDA level. (B) Effect of quercetin on clivorine-increased GSH level in the liver. Data are expressed as means ± SEM (n = 9 to 10). ***P<0.001 compared with the control; *^#^P<0.05, ^##^P<0.01* compared with clivorine.

**Figure 6:**
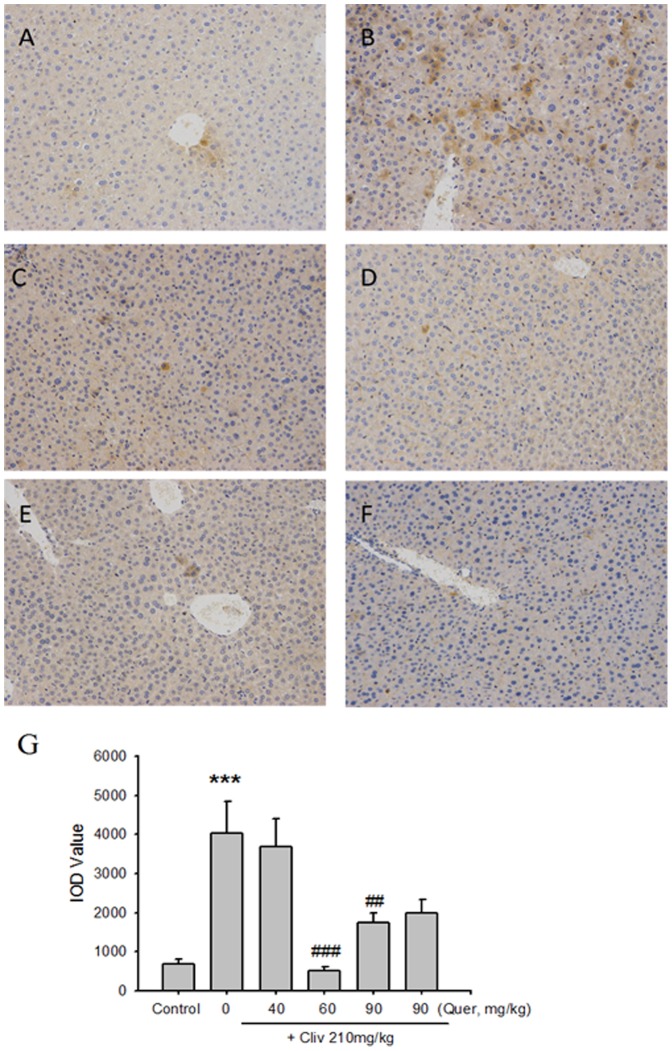
Liver immunohistochemical analysis of 4-HNE. Typical images were selected from each experimental group (original magnification: 200×). (**A**) Vehicle control, (**B**) clivorine (210 mg/kg), (**C**) clivorine (210 mg/kg) + quercetin (40 mg/kg), (**D**) clivorine (210 mg/kg) + quercetin (60 mg/kg), (**E**) clivorine (210 mg/kg) + quercetin (90 mg/kg), and (**F**) quercetin (90 mg/kg). (**G**) IOD values of 4-HNE staining were counted manually in at least three random fields every section. Data are expressed as means ± SEM (n = 6). *P<0.05 compared with the control; *^#^P<0.05* compared with clivorine.

Clivorine (210 mg/kg) increased the MDA and GSH levels in the liver (P<0.001), but quercetin (90 mg/kg) reversed such increase (P<0.01) (Figs. 5A and B). Quercetin (60 mg/kg) also decreased clivorine-induced increase in the GSH level (P<0.05). Liver immunostaining of 4-HNE, which is a marker for LPO, was weak in the control and quercetin (90 mg/kg)-treated mice (Figs. 6A and F). The 4-HNE staining cells increased in clivorine-treated mice (Fig. 6B). Quercetin (60 and 90 mg/kg) reduced clivorine-increased 4-HNE staining cells (Figs. 6D and E). The statistical result in Fig. 6G demonstrated that clivorine enhanced the immunohistochemical staining of 4-HNE, contrary to the evident quercetin (60 and 90 mg/kg)-induced reduction in clivorine-increased 4-HNE staining (P<0.01, P<0.001).

### Results of mouse stress & toxicity pathway Finder PCR array

**Figure 7:**
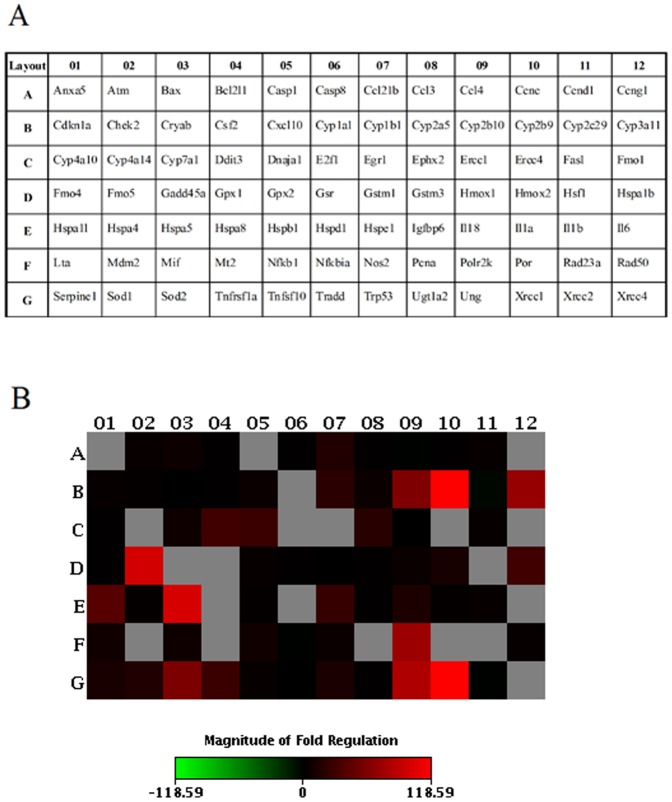
Expression profile of 84 genes included in the Stress and Toxicity PathwayFinder PCR Array in control and quercetin-treated mice. (A) The layout of the genes included in the Stress and Toxicity PathwayFinder PCR Array. (B) Heat map of the variations in the expression of 84 genes between control and quercetin-treated mice are shown as a fold increase or decrease.

The layout of the genes of PCR array is shown in Fig. 7A, and Fig. 7B is a heat map that shows the fold regulation expression data between quercetin (90 mg/kg) and the control group. Numerous red and purple data are shown in Fig. 7B, which indicates that the expression of such genes was up-regulated in the livers of quercetin-treated mice. The differentially expressed genes over 5-folds between control and quercetin-treated group are listed in Table 1. Fmo5, Polr2k, Sod2, Ephx2, Sod1, Hmox2, and Hmox1 belong to the family of oxidative stress. Cyp2b10, Cyp1b1, Cyp2a5, Cyp3a11, and Cyp7a1 belong to the family of metabolic stress. Hspa5, Hspa1l, Hspa1b, Dnaja1, and Hspe1 belong to the family of heat shock.

**Table 1:** Variation in the liver gene expression between control and quercetin (90 mg/kg)-treated mice in RT^2^ Profiler PCR array.

Functional Gene Grouping	Gene Bank	Symbol	Protein	Up-regulation or Down-regulation Quer/Cont
Oxidative or Metabolic Stress	NM_009999	Cyp2b10	Cytochrome P450, family 2, subfamily b, polypeptide 10	59.00
	NM_009994	Cyp1b1	Cytochrome P450, family 1, subfamily b, polypeptide 1	20.38
	NM_007812	Cyp2a5	Cytochrome P450, family 2, subfamily a, polypeptide 5	5.49
	NM_010000	Cyp2b9	Cytochrome P450, family 2, subfamily b, polypeptide 9	119.04
	NM_007818	Cyp3a11	Cytochrome P450, family 3, subfamily a, polypeptide 11	71.8
	NM_007824	Cyp7a1	Cytochrome P450, family 7, subfamily a, polypeptide 1	7.69
	NM_007940	Ephx2	Epoxide hydrolase 2, cytoplasmic	19.27
	NM_010232	Fmo5	Flavin containing monooxygenase 5	99.31
	NM_010442	Hmox1	Heme oxygenase (decycling) 1	5.23
	NM_010443	Hmox2	Heme oxygenase (decycling) 2	10.76
	NM_011434	Sod1	Superoxide dismutase 1, soluble	15.37
	NM_023127	Polr2k	Polymerase (RNA) II (DNA-directed) polypeptide K	74.25
	NM_013671	Sod2	Superoxide dismutase 2, mitochondrial	58.16
Heat Shock	NM_008298	Dnaja 1	DnaJ (Hsp40) homolog, subfamily A, member 1	27.60
	NM_010478	Hspa1b	Heat shock protein 1-like	30.65
	NM_013558	Hspa1l	Heat shock protein 1-like	40.74
	NM_008303	Hspe1	Heat shock protein 1 (chaperonin 10)	25.61
	NM_022310	Hspa5	Heat shock protein 5	101.43

Cont: Control; Quer: Quercetin (90 mg/kg).

### Results of real-time PCR analysis

Real-time PCR was used to further verify the up-regulated genes in the previous PCR array. In addition, we also observed whether clivorine altered the expression of those above genes and the potential effects of quercetin.

**Figure 8:**
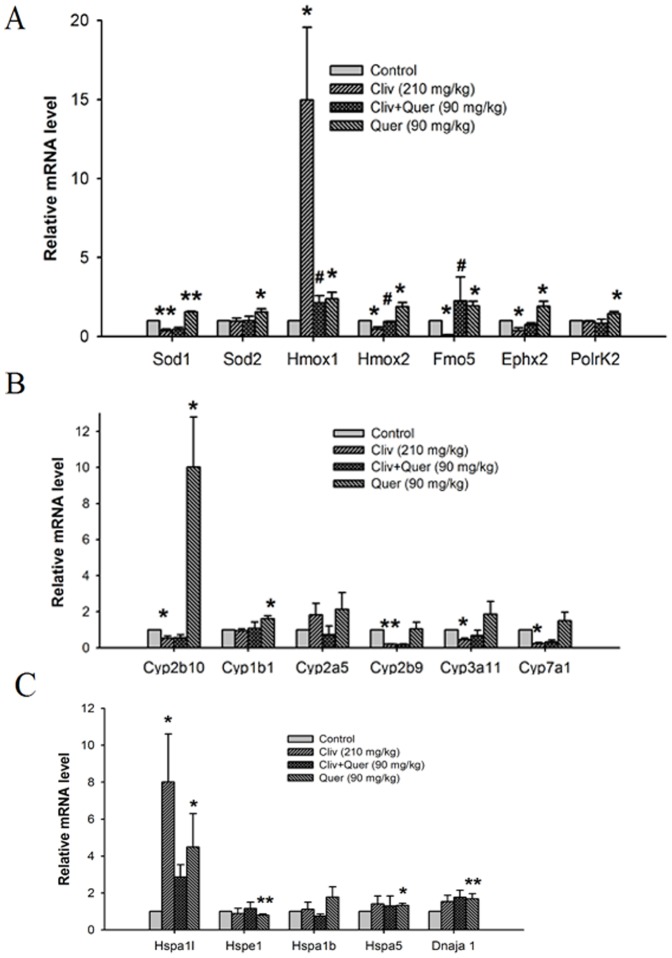
Validation of gene expression by real-time PCR analysis. The mRNA expression of (A) Fmo5, Polr2k, Sod2, Ephx2, Sod1, Hmox2, and Hmox1; (B) Cyp2b10, Cyp1b1, Cyp2a5, Cyp3a11, and Cyp7a1; and (C) Hspa5, Hspa1l, Hspa1b, Dnaja1, Hspe1. Data are expressed as means ± SEM (n = 6 to 8). *P<0.05, ***P<0.001 compared with the control.

The expression of Fmo5, Polr2k, Sod2, Ephx2, Sod1, Hmox2, and Hmox1 genes was up-regulated in the livers of the quercetin-treated mice, which further confirmed the previous results in the PCR array (P<0.001, P<0.05) (Fig. 8A). In addition, clivorine decreased the mRNA expression of Sod1, Hmox2, Fmo5, and Ephx2 (P<0.05, P<0.01), whereas quercetin (90 mg/kg) reversed the decreased expression of Hmox2 and Fmo5 (P<0.05). Clivorine increased the mRNA expression of Hmox1 (P<0.05), whereas quercetin (90 mg/kg) decreased such increase (P<0.05).

Cyp2b10 expression was highly up-regulated (P<0.05), but Cyp1b1 expression was weakly up-regulated (P<0.05), in quercetin-treated mice (Fig. 8B). However, no obvious up-regulation was found in Cyp3a11, Cyp2b9, Cyp2a5, and Cyp7a1 expression in quercetin-treated mice, which was inconsistent with the results of the previous PCR array. In addition, clivorine decreased the expression of Cyp2b10, Cyp2b9, Cyp3a11, and Cyp7a1 (P<0.05, P<0.01). After treatment with quercetin (90 mg/kg), the decreased expression of all those above genes was not reversed by quercetin.

Liver Hspa1l, Hspa5, and Dnaja1 expression was up-regulated in quercetin-treated mice (P<0.05) (Fig. 8C), and no evident up-regulation was found in the Hspa1b expression in quercetin-treated mice. However, Hspe1 expression was down-regulated in quercetin-treated mice (P<0.05), which is inconsistent with the previous PCR array results. In addition, clivorine increased the expression of Hspal1 (P<0.05), and although quercetin has the tendency to decrease the increased Hspal1 expression, but there is no statistical significance (P>0.05).

## Discussion

Drug-induced liver injury (DILI) is an important public health problem affecting thousands of people worldwide. Chinese herbal medicine is traditionally considered as having no obvious toxicity and side effect. With the commonly used herbal and dietary supplements, liver toxicity and side effect induced by herbal medicines are concomitantly reported and has aroused widespread concern. PA is a type of natural hepatotoxin distributed in a variety of plants, and PA-containing plants are the most common poisonous plants that affect livestock and humans because of their global distribution. Over 8000 cases of PA-induced hepatic sinusoidal obstruction syndrome (HSOS), previously called hepatic veno-occlusive disease, have been reported globally [18]. However, no strategy has been formulated on the detoxification of PA-induced hepatotoxicity.

Our results demonstrate that quercetin can prevent PA clivorine-induced acute liver injury in mice as indicated by the results from the analysis of serum ALT, AST enzymatic activity, TB level, and histological assessment. Quercetin is a well-known natural antioxidant and a commonly used flavonoid. Several herbal medicines, including *Senecio cannabifolius var. integrilifolius* and *Gynura segetum*, contain quercetin [19], [20]. These two herbal medicines also contain a considerable amount of PA [18], [21]. *G. segetum*, also called “Tusanqi” in Chinese medicine, was reported to be the major causes of HSOS in clinic when PA-containing herbal medicines are consumed [18]. Thus, our results not only provide the potential strategy for the detoxification of PA-induced liver injury, but also evidence for the advantage of Chinese medicines considering that the medicinal plant is entirely consumed but not as individual compounds, which will lead to the possibility of PA detoxification caused by the other compounds contained in Chinese medicines.

The mechanism of the pathogenesis of DILI has been extensively investigated, in which apoptotic cell death, oxidative or metabolic liver injury, and inflammation are reported to be involved in the process of DILI [22]–[25]. We found in our previous reports that PA clivorine can induce apoptotic cell death and oxidative stress injury in human normal liver L-02 cells [7], [8]. The present results of TUNEL assay and Western-blot analysis of caspase-3 demonstrate that quercetin prevented clivorine-induced apoptotic cell death. Liver MDA levels and immunohistochemical staining of 4-HNE reflect that quercetin can prevent clivorine-induced LPO. GSH is an important endogenous antioxidant, and its homeostasis has been associated with liver injury induced by drugs, alcohol, and other exogenous toxins [26], [27]. The present results show that clivorine induced the increase in cellular GSH level, which may be caused by the elevation of the body defense capacity to defend clivorine-induced oxidative stress injury. After quercetin treatment, the increased liver GSH level was reduced, which suggests that oxidative stress injury was ameliorated.

Further, the expression profile of 84 genes included in the Stress and Toxicity PathwayFinder PCR Array demonstrated that oxidative or metabolic stress-related genes, including Fmo5, Polr2k, Sod2, Ephx2, Sod1, Hmox2, Hmox1, Cyp2b10, Cyp1b1, Cyp2a5, Cyp3a11, and Cyp7a1, and heat shock-related genes, including Hspa5, Hspa1l, Hspa1b, Dnaja1, and Hspe1, were up-regulated in quercetin-treated mice compared with control. The results indicate the potential involvement of those signals in regulating the protection of quercetin against clivorine-induced liver injury.

The up-regulated expression of Fmo5, Polr2k, Sod2, Ephx2, Sod1, Hmox2, and Hmox1 induced by quercetin was further confirmed by the real-time PCR analysis. In addition, clivorine-decreased expression of Hmox2 and Fmo5 was reversed by quercetin. Sod2, Ephx2, Sod1, Hmox2, Hmox1, and Fmo5 encode critical proteins or antioxidant enzymes involved in regulating cellular redox homeostasis [28]–[31]. A previous report has already demonstrated that flavin-dependent monooxygenase (FMO) in arctiid moth *Tyria jacobaeae* is critical for PA detoxification, and there are five members of FMO (FMO1-5) found in mammals [32]. The present study demonstrates that quercetin increased Fmo5 gene expression, and it also increased clivorine-decreased Fmo5 expression, which may contribute to the detoxification of quercetin against PA clivorine. The results further prove that quercetin can prevent clivorine-induced liver oxidative stress injury by elevating antioxidant capacity.

CYP450, especially members of CYP3A family, is related with the metabolic activation of various PAs such as senecionine, monocrotaline etc [33]–[36]. The up-regulated expression of Cyp2b10 and Cyp1b1 induced by quercetin was confirmed by the real-time PCR analysis. In addition, clivorine decreased the mRNA expression of Cyp2b10, Cyp2b9, Cyp3a11, and Cyp7a1, but quercetin had no effect on clivorine-decreased expression of those genes. Our previous studies have demonstrated that clivorine can induce cytotoxicity in human normal liver L-02 cells and kidney HEK293 cells, and those two cells are lack of CYP450 enzymes [4], [37], which indicates that metabolic activation is not request for the hepatotoxicity induced by clivorine. In addition, there is no report about the involvement of Cyp2b10 or Cyp1b1 in the metabolic activation of pyrrolizidine alkaloids. Our results indicate that the protection of quercetin against clivorine-induced liver injury may not attribute to the increased expression of Cyp2b10 or Cyp1b1.

Under sublethal stress, cellular heat shock proteins will be transiently overproduced and thereby develop tolerance to the stress injury, which is also related with the outcome of stress liver injury [38]. The up-regulated expression of Hspa1l, Hsp5, and Dnaja 1 was further confirmed by real-time PCR analysis. In addition, clivorine also increased the mRNA expression of Hspa1l. The present results indicate that quercetin can increase the capacity of body to defend trauma or stress injury induced by clivorine, and of which Hspa1l may exert important role.

## Conclusion

The present study demonstrates that quercetin can prevent PA clivorine-induced acute liver injury *in vivo* by inhibiting apoptotic cell death and ameliorating oxidative stress injury. Quercetin can enhance the body defense capacity to prevent clivorine-induced hepatotoxicity by regulating the expression of genes related with oxidative homeostasis or heat shock. Our study provides the strategy and reference for the detoxification of PA-induced liver injury.

## Supporting Information

Table S1 **List of the used primers in Real-time PCR experiment.**
(DOCX)
