# Supplementary material for: Quercetin Prevents Pyrrolizidine Alkaloid Clivorine-Induced Liver Injury in Mice by Elevating Body Defense Capacity
Source: PLoS One. 2014 Jun 6;9(6):e98970. doi: 10.1371/journal.pone.0098970 (PMC4048295; doi:10.1371/journal.pone.0098970)
Supplement: Table S1 — List of the used primers in Real-time PCR experiment. (DOCX) [file pone.0098970.s001.docx]

**Supplementary Tab.1** List of the used primers in Real-time PCR experiment

|  | Primer | Sequence |
| --- | --- | --- |
| Sod1 | FP | 5’-CCAGCATGGGTTCCACGTCCAT-3’ |
|  | RP | 5’-TCCGCCGGGCCACCATGTTT-3’ |
| Sod2 | FP | 5’-CCACGCGGCCTACGTGAACA-3’ |
|  | RP | 5’-TGCAGGCTGAAGAGCGACCTG-3’ |
| Hmox1 | FP | 5’-TGACACCTGAGGTCAAGCAC-3’ |
|  | RP | 5’-GTCTCTGCAGGGGCAGTATC-3’ |
| Hmox2 | FP | 5’-GAGTGAGGGCAGCACAAACTA-3’ |
|  | RP | 5’-GAGCTCAGAAAGGTCTGCCAT-3’ |
| Fmo5 | FP | 5’-CAAGGACGCTGGGCCACTCAA-3’ |
|  | RP | 5’-CCTGGATGGTATGACGCTGGCT-3’ |
| Ephx2 | FP | 5’-CCTTCCAGCTTCGTGTCTGT-3’ |
|  | RP | 5’-TCCCTCTGGGAATTCCGTCT-5’ |
| Polrk2 | FP | 5’-TTGTGGAGAGTGTCACACCG-3’ |
|  | RP | 5’-ACTCCCACATTTCATCGAGCA-3’ |
| Cyp2b10 | FP | 5'-ACGTTCCTCTTCCAGTGCATCA-3' |
|  | RP | 5'-CTGGCTGGAGAATGAGCTTATGAG-3' |
| Cyp1b1 | FP | 5’-GTGCCTGCCACTATTACGGACAT-3’ |
|  | RP | 5’-TCCCCAAACCTGGTCCAAC-3’ |
| Cyp2a5 | FP | 5'-ACGGTATTGTTTCGGAGAAGGACT-3' |
|  | RP | 5'-AGTGTAGGTTGGTGGGATCGTG-3' |
| Cyp2b9 | FP | 5’-GCCTTCTTAAGTGTTTCATTCAGCT-3’ |
|  | RP | 5’-CACCAGAGCCTCCCTTATCGTC-3’ |
| Cyp3a11 | FP | 5’-GAACTTCTCCTTCCAGCCTTGTAAG-3’ |
|  | RP | 5’-CGTGGCACAACCTTTAGAACAAT-3’ |
| Cyp7a1 | FP | 5'-CTTCATCACAAACTCCCTGTCATAC-3' |
|  | RP | 5'-TGGTATTTCCATCACTTGGGTCTAT-3' |
| Hspa1l | FP | 5’-TCCAAACTGGATCGAAGGCG-3’ |
|  | RP | 5’-CTGGTCGTTGGCGATGATCT-3’ |
| Hspe1 | FP | 5’-GGCGAAGGCGAGAGTCAT-3’ |
|  | RP | 5’-CCAACTTTCACACTGACAGGC-3’ |
| Hspa1b | FP | 5’-TCTGCTGGCTCTCCCGGTGTG-3’ |
|  | RP | 5’-ACCTTGACAGTAATCGGTGCCCAA-3’ |
| Hspa5 | FP | 5’-CACGGTGGTCGGCATCGACT-3’ |
|  | RP | 5’-TACGACGGCGTGATGCGGTT-3’ |
| Dnaja 1 | FP | 5’-CAGGGTCATGGAGAACGCAT-3’ |
|  | RP | 5’-GAAGCCGCACAATGCTTCAA-3’ |
| Actin | FP | 5'-TTCGTTGCCGGTCCACACCC-3' |
|  | RP | 5'-GCTTTGCACATGCCGGAGCC-3' |

FP, Forward Primer; RP, Reverse Primer
